# Magnetic excitation of a granular gas as a bulk thermostat

**DOI:** 10.1038/s41526-019-0079-y

**Published:** 2019-08-13

**Authors:** Masato Adachi, Peidong Yu, Matthias Sperl

**Affiliations:** 1 Institut für Materialphysik im Weltraum, Deutsches Zentrum für Luft- und Raumfahrt (DLR), 51170 Köln, Germany; 2 Institut für Theoretische Physik, Universität zu Köln, 50937 Köln, Germany

**Keywords:** Techniques and instrumentation, Statistical physics, thermodynamics and nonlinear dynamics

## Abstract

A thermostat utilizing a varying magnetic field has been developed to agitate soft ferromagnetic particles in microgravity platforms for an investigation of an energy-dissipative granular gas. Although the method has experimentally realized a reasonably homogeneous spatial distribution of particles, the physics behind the magnetically excited particles has not been understood. Therefore, a numerical calculation based on the discrete element method is developed in this paper to explain the realization of homogeneously distributed particles. The calculation method allows considering inelastic and magnetic interactions between particles and tracking the motions due to those interactions during the excitation of the granular gas. The calculation results, compared with the experimental result, show that magnetic interactions between particles, a time-domain variation of magnetic-excitation directions, and random collisions of particles between each magnetic excitation contribute to distribute particles homogeneously.

## Introduction

A granular gas is a dilute system of macroscopic particles where the constituent particles collide with each other, causing dissipation of energy.^1^ Investigations of the granular physics during the energy-loss process require realizing two challenging conditions: (i) removal of gravitational force acting on particles to avoid sedimentation, and (ii) homogeneous distribution of particles in microgravity by a proper external excitation similar to a thermostat for a molecular gas system.^1–3^ Dissipative granular gases have been investigated in depth by simulations,^4–10^ because the ideal spatial distribution and random excitations are readily available.

On the other hand, experimental investigations have been carried out in various kinds of microgravity platforms to meet condition (i) (e.g., drop tower, parabolic flights of an aircraft, and sounding rocket), while the particles were excited by conventional ways, such as boundary shaking, to meet condition (ii).^11–18^ In addition, other excitations using magnetic forces have been developed.^2,19,20^ Unlike boundary shaking, magnetic-excitation forces are long range and applied to all the particles within a sample cell, resulting in a reasonably homogeneous spatial distribution of particles excited randomly. There are several ways of applying the magnetic force, such as diamagnetic levitation of magnetic particles using a superconducting magnet,^19^ and the use of Helmholtz coils and permanent magnets encased in glass cylinders.^20^ The behaviors of those particles during the excitations have been investigated. The former system allows shaking particles on the ground by using an additional coil, while gravity is compensated by the diamagnetic levitation. The latter system exerts magnetic torques on the cylindrical particles in a uniform magnetic field and prevents interactions between the magnets by using the casing. As for the prevention of interactions between permanent magnets, an assembly of eight spherical magnets into one particle with a dotriacontapole configuration is also available.^21^ Interactions between the magnets placed optimally makes the magnetic field decaying with the inverse seventh power of the distance outside the assembly, realizing no clustering between the assemblies.

**Fig. 1:**
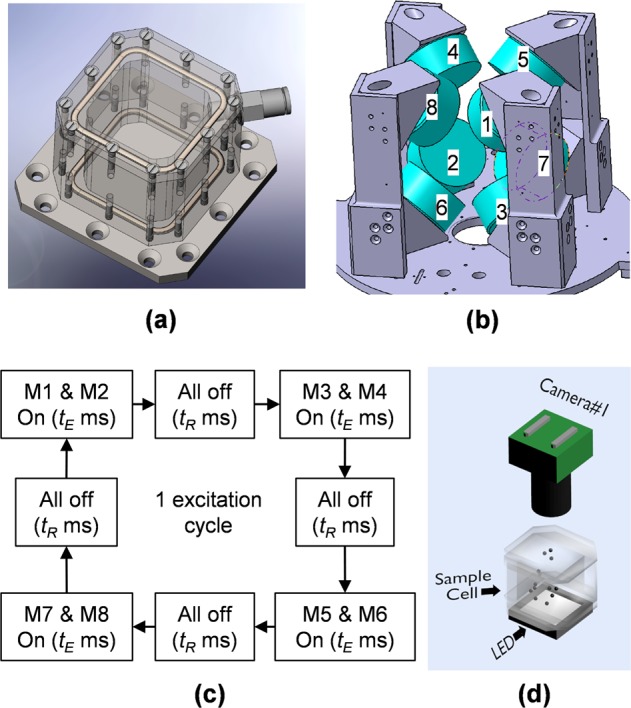
Schematics of the experimental setup. The setup consists of **a** a sample cell and **b** eight electromagnets. The electromagnets are operated in **c** an excitation sequence to agitate particles (*t*_*E*_: turning-on time of one pair of electromagnets, *t*_*R*_: turning off of all electromagnets), and the motion of the particles is observed by using **d** an imaging system. (**a**–**d**: Republished with permission of AIP Publishing from Yu et al.;^2^ permission conveyed through Copyright Clearance Center, Inc.)

As an alternative, magnetic excitation employing soft ferromagnetic particles with high magnetic permeability and a varying magnetic field was also realized on a sounding rocket that offers a long low-gravity duration (375 s) along with a very low remnant gravity level (~10^−^^5^ g).^2^ The spherical particles (number: 3000, radius: 0.8 mm, relative magnetic permeability *μ*_max_: 4.5 × 10^4^, and mumetal, Sekels GmbH) are placed in the sample cell (Fig. 1a, inner dimension: 5 × 5 × 5 cm^3^), and the packing fraction is ~5%, within the Knudsen regime. During the experiment, the inner space of the cell connects to the outer space of the rocket, which has a low air pressure of about 10 Pa. The effect of air drag is thus negligible.^2^ The particles are excited in external magnetic fields provided by eight commercial electromagnets (Fig. 1b). The magnets are placed near the eight corners of the cubic cell and face each other along the diagonal lines of the cell. When turned on, the magnet closest to a particle attracts this particle toward the corresponding corner. A time sequence of the excitation is thus needed to sustain the motion of the particles (Fig. 1c). At one point in time, only two magnets facing against each other are turned on, while the others remain off. At the next phase, another pair of magnets is activated to drag the particles to other directions. By using four pairs of these excitation sources positioned in 3D, a reasonably uniform 3D spatial distribution of the particles is achieved. The motion of the particles is captured by using a light-field camera (Fig. 1d, Raytrix R5), and 3D information can be retrieved from the experiment. This method meets conditions (i) and (ii) because the granular material has such high permeability. The particles have fast response to an external magnetic field, as well as low remnant magnetization, as soon as the field is turned off. The former property ensures that enough particles (~3000) can be thoroughly excited within a short time period in microgravity environments, while the latter ensures negligible particle–particle magnetic long-range interaction during the cooling. In the experiment performed in microgravity, a homogeneous distribution of the ferromagnetic particles was realized by this setup.^2^

However, the observation is in apparent contradiction to the common behavior of magnetized particles, which conventionally prefer creating their own chains due to magnetic dipole moments in the particles, aligning in the same direction of an external magnetic field and preventing the homogeneous distribution. Magnetic interactions between the particles have been studied mainly in industrial fields, such as electrophotography process.^22^ Static and dynamic states of magnetic particle chains during the process have been investigated by experiments and simulations,^23–25^ and mechanical and magnetic properties of the chains were analyzed in detail, as well as the chain-growing mechanism. However, these previous works are based on the case when magnetic particles are attracted toward permanent magnets, but not on the case when particles are excited from various directions by varying the magnetic field. Thus, the physics behind a dilute granular gas consisting of soft ferromagnetic particles has not been investigated thoroughly.

To investigate the magnetically excited granular gas, a numerical calculation is developed in this paper based on the discrete element method (DEM).^26^ The DEM can consider inelastic and electromagnetic interactions between particles. The method has been used in many granular research fields.^27–31^ Our simulation serves (1) to help evaluate and explain the existing experimental result of a granular gas system,^2^ and (2) to offer a way of predicting the behavior with different parameters to improve such thermostat for future experiments. The calculation method has the capability of considering interactions between magnetic dipole moments created in particles which are subjected to the external magnetic field. The details of the simulation process can be found in the section “Methods” at the end of the paper. Using the simulation, we calculate the motion of particles, which are agitated by the magnetic thermostat, in cases of neglecting magnetic particle–particle interactions (case A) and including the interactions (case B). We compare the simulation results with the experiment performed in microgravity.^2^ Our major finding from the comparison is the vital contribution by the magnetic interactions between the particles to realize the homogeneous distribution.

## Results

**Fig. 2:**
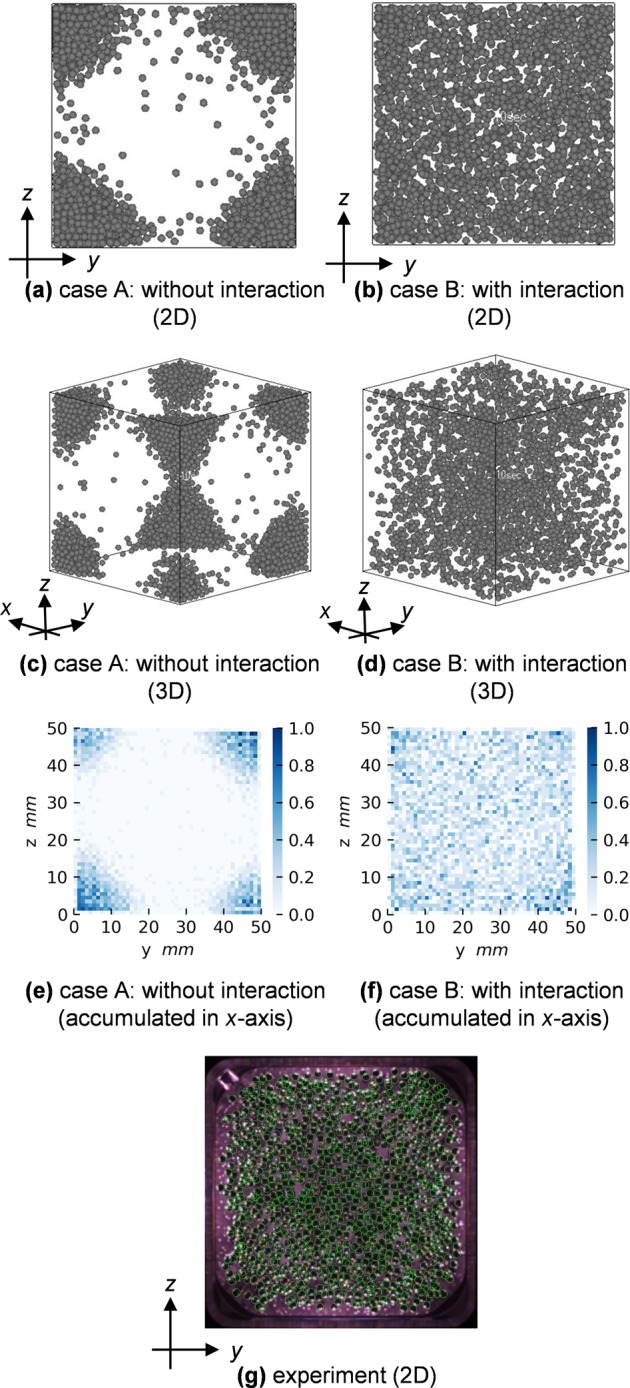
Calculated and observed motions of agitated particles. Snapshots of calculated particles excited in **a**, **c** case A (without magnetic interactions) and **b**, **d** case B (with magnetic interactions) are taken at the end of rounds of magnetic excitations (one magnetic excitation: 20 ms on and 80 ms off) for 10 s in low g. The particles are displayed in **a**, **b** 2D and **c**, **d** 3D. Spatial distributions of the particle densities, accumulated in the *x* direction, are shown in **e** case A and **f** case B. **g** A snapshot of experimentally agitated particles in low g is shown with green line contours for outlining the particles. (**g**: Republished with permission of AIP Publishing from Yu et al.;^2^ permission conveyed through Copyright Clearance Center, Inc.)

Figure 2a–d shows calculated particle motions, in cases of neglecting magnetic particle–particle interactions (case A) and including the interactions (case B) (see Supplementary Movies 1 and 2). In addition, Fig. 2e, f shows spatial distributions of particle densities in the same configurations. Figure 2 shows qualitatively that the particles are distributed more homogeneously in case B, and the 2D snapshot (Fig. 2b) resembles closely the experiment (Fig. 2g, see Supplementary Movie 3). In case A, the particles prefer moving toward corners of the sample cell, as shown in Fig. 2c, e. One may assume that during the experiment, particle–particle magnetic interactions are undesired.^2^ This assumption is true during the cooling phase, and the interactions are indeed avoided by using the ferromagnetic particles with very low remnant magnetization. During excitation phases, the particle chain structure induced by the interaction may disturb the desired spatial homogeneity. However, the comparison of the two cases shows that the particle–particle interaction favors the desired homogeneous state. The detailed mechanism is explained in the section “Discussion”.

**Fig. 3:**
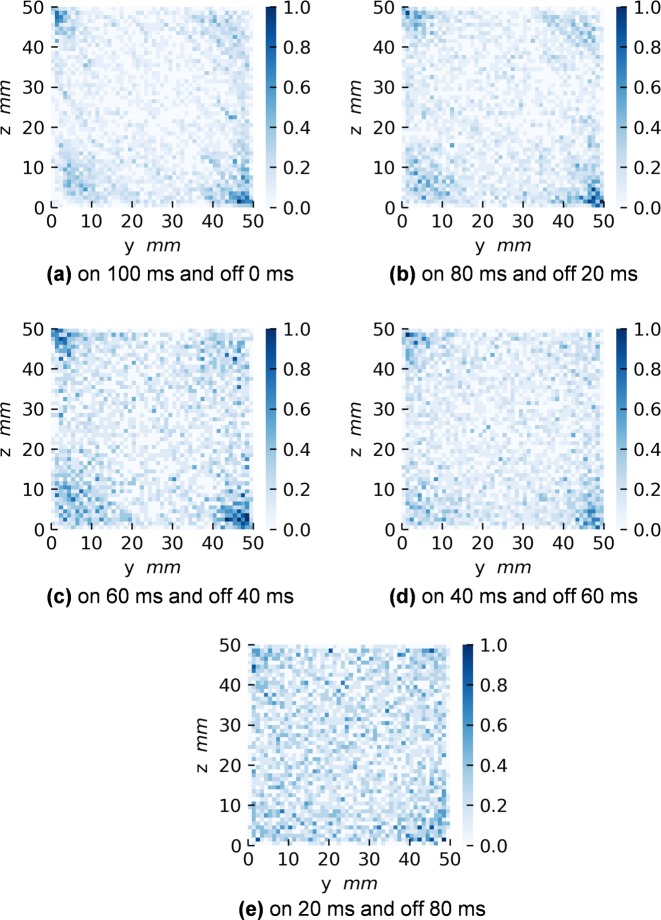
Spatial distributions of particle densities at the end of excitations in case B (with magnetic interactions). The densities are accumulated in the *x* direction, when the excitation duration is varied, such as **a** 100 ms on and 0 ms off, **b** 80 ms on and 20 ms off, **c** 60 ms on and 40 ms off, **d** 40 ms on and 60 ms off, and **e** 20 ms on and 80 ms off

Because durations of turning on and off electromagnets are crucial for the spatial homogeneity of particles,^2^ those effects were investigated. Figure 3 shows spatial distributions of particle densities at the end of rounds of magnetic excitations, when the excitation duration is varied in case B. Although the distribution is more homogeneous in all the results (Fig. 3a–e) than that in case A (Fig. 2e), the particles tend to move toward the corners of the sample cell when the excitation duration is increased. In the experiment,^2^ the most favorable values to get the homogeneous distribution were the magnetic excitation of 20 ms on and 80 ms off, which are the same as those in the simulation results. The desired experimental values were found after a series of experiments in microgravity reproduced by the different platforms, i.e., drop tower, parabolic flight, and sounding rocket.^2^ The numerical calculation can now be used to investigate the optimal parameters before the experiments.

**Fig. 4:**
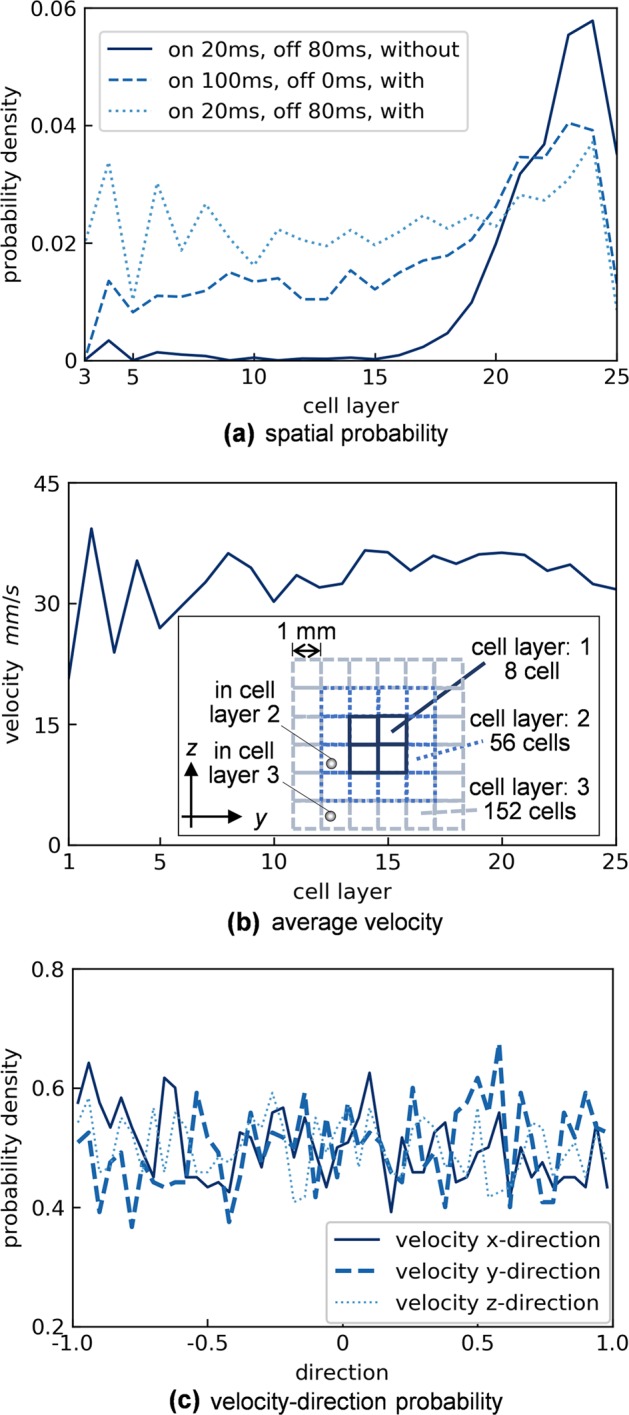
Spatial and velocity homogeneity of particles agitated by magnetic excitations. **a** Calculated number densities and **b** a calculated average velocity of the particles existing in each cell layer. **c** Calculated particle-velocity-direction probabilities in each axis. A virtual cell layer is used in **a**, **b** to sort particles spatially in terms of the distance from the center of the sample cell, as shown in **b**, because the sample cell shape is not spherical, and it is convenient to apply a cubic cell leaving no space within the whole sample cell. Each virtual cell has 1-mm length on all the sides, and the sample cell (50 × 50 × 50 mm^3^) is divided into 50 × 50 × 50 cells. The data are obtained at the end of the magnetic excitations, and those in **b**, **c** are taken in the optimal condition (with interaction, 20 ms on and 80 ms off for 10 s)

In the optimal configuration (20 ms on and 80 ms off), spatial and velocity homogeneity of the distribution were evaluated quantitatively by measuring a number density of particles and an average velocity with respect to distance from the center of the sample cell, respectively, as shown in Fig. 4a, b. In addition, Fig. 4c shows a particle-velocity-direction probability. Figure 4a, b shows that the particles are distributed more homogeneously in the optimal case, and the velocity magnitude is almost uniform wherever the particles are located. Moreover, the particles move randomly and almost omnidirectionally at the end of using this thermostat, as shown in Fig. 4c. In Fig. 4a, although the particles are distributed almost homogeneously in the optimal case, compared with those in different conditions, a preference of particle location is still near the corners of the sample cell, due to the geometric effect. It can be improved by optimizing the geometry of the sample cell and the way of applying the magnetic field. The improvements shall be tested in future experiments on microgravity platforms.

**Fig. 5:**
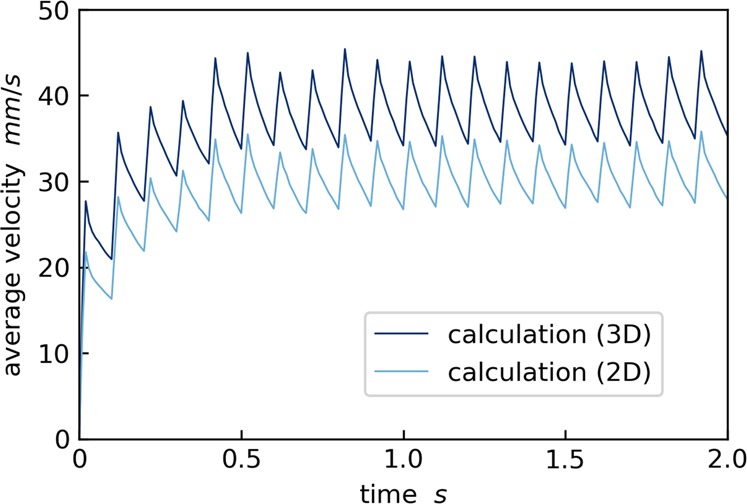
Calculated average velocities of particles during the beginning of the excitations. The velocity variations as a function of time are displayed in 2D (the *y-* and *z-*axis plane) and 3D. The particles are excited by the magnetic field of 20 ms on and 80 ms off

Figure 5 shows the calculated average velocities of the excited particles with respect to time. The frequency of the velocity fluctuation corresponds to the timing of switching between electromagnet pairs. As shown in Fig. 5, the average velocities are almost saturated 1 s after starting the excitations. In the experiment,^2^ homogeneously distributed particles were also realized shortly after initiating magnetic agitations. Therefore, this thermostat does not require a long duration to obtain the homogeneous distribution. In addition, the average velocity in 2D is obviously smaller than that in 3D. The velocity ratio between 2D and 3D should be 1.225 theoretically for an ideal gas, but the ratio of the calculation is almost that value. The calculation allows obtaining the average velocities in 3D, which is hard to measure in the experiments, due to the overlapping of particles.^2^ The simulation has also a potential to investigate behavior of the freely cooling particles after the magnetic excitation. The cooling phase of a granular gas has been investigated in depth by simulations, such as an instability of clustering with consideration of the kinetic gas theory,^1,4–6^ and an exchange of translational and rotational energies of particles,^7–10^ and deviations from the Boltzmann behavior. The further investigation of the cooling behavior of the granular gas driven by this thermostat shall be conducted in future work.

In brief, the simulation results reveal that the magnetic interactions between particles contribute in a significant way to the realization of the homogeneous distribution. The simulation can reproduce the experiment, and it allows analyzing a granular gas in the excitation phase and potentially the subsequent cooling phase in detail.

## Discussion

**Fig. 6:**
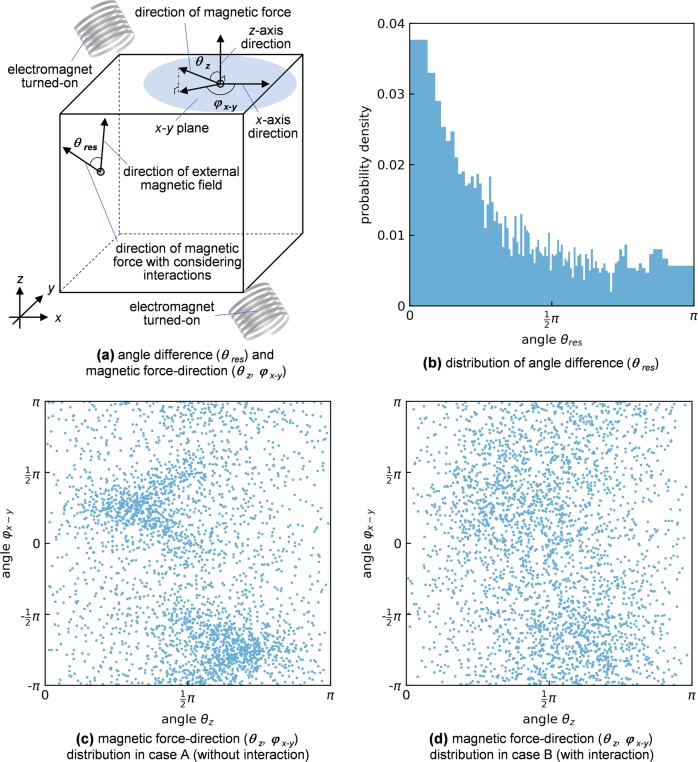
Magnetic force–direction distributions. **a** Definitions of an angle difference (***θ***_res_) between magnetic force–directions in cases A (without interaction) and B (with interaction) and a magnetic force angle in the fixed coordinate system in lab frame (***θ***_*z*_, ***φ***_*x−y*_). The ***θ***_res_ shows a deviation of the force–direction affected by the magnetic interactions between particles. An external magnetic field–direction at a particle position is the same as a direction of the magnetic force applied to the particle in case A. The ***θ***_res_ is the difference between the external magnetic field–direction and the magnetic force–direction in case B. The ***θ***_*z*_ is determined as a zenith angle from the *z* axis, and the ***φ***_*x−y*_ is used as an azimuth angle from the *x* axis. **b** Distributions of the angle difference (***θ***_res_), and **c**, **d** the magnetic force–directions in case A and case B (***θ***_*z*_, ***φ***_*x−y*_). The magnetic forces applied at the first round of magnetic excitations are analyzed (at 0.01 s), and at the state, the particles move for inappreciable distances since the initial state in which the particles are distributed randomly

As shown in the section “Results”, case B (with magnetic interactions) is more effective than case A (without magnetic interactions). To understand the mechanism of realizing a homogeneous distribution of magnetic particles, magnetic force–direction distributions during the excitations are shown in cases A and B (Fig. 6). Here, two types of variables for analyzing the force–direction are used, as shown in Fig. 6a: (I) one is an angle difference between magnetic force–directions in cases A and B (***θ***_res_), and (II) the other is a magnetic force angle in the fixed coordinate system in the lab frame (***θ***_*z*_, ***φ***_*x−y*_). The former variable (I) shows a deviation of the force–direction affected by the magnetic interactions between particles. The latter variable (II) is for showing a homogeneity of magnetic force–direction in a consistent evaluation way. Figure 6b shows that the angle difference ***θ***_res_ ranges all the way from 0 to π, with the most populated direction (***θ***_res_ = 0). In other words, although the magnetic force direction in case B still tends to face toward the field direction (***θ***_res_ = 0), the magnetic interactions between particles largely affect the force to the extent that the force can be totally flipped (***θ***_res_ = π). Figure 6c shows that there are two preferred directions of the magnetic field facing toward two electromagnets turned on in case A, while the direction preference is alleviated in case B, as shown in Fig. 6d. That is because magnetic dipole moments in particles affect each other and are prone to balance, and it makes the force–direction and even the spatial distribution more homogeneous. Moreover, Fig. 6 shows that only one time frame is taken when one pair of two electromagnets are turned on, and the force–direction will be further randomized in a long term when switching from pair to pair of the electromagnets.

**Fig. 7:**
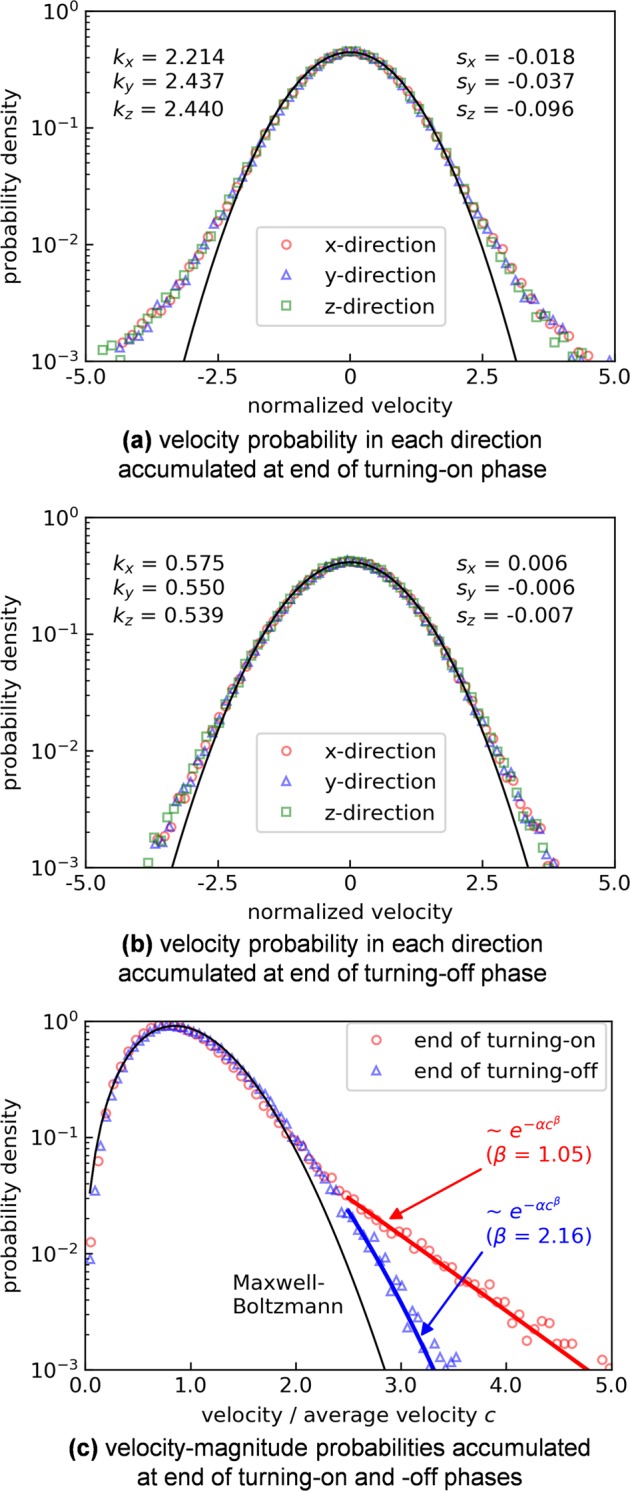
Velocity probabilities of agitated particles. **a**, **b** Normalized velocity probabilities in each *x-, y-*, and *z* direction at the end of the excitation phase (20 ms) and at the end of the non-excited phase (80 ms), respectively, and **c** velocity-magnitude probabilities. The particle velocities measured at 50 different frames are accumulated for a better statistic for both cases (e.g., turning on: at 0.02, 0.12, 0.22 s…, turning off: at 0.1, 0.2, 0.3 s…). One time frame data contain 3000 number of particles, and data accumulated for 50 time frames, which contain 150,000 number of particles, are utilized statistically. In **a**, **b**, Gaussian fits are drawn, and the skewness *s* and the kurtosis *k* (subtracted for 3) of the distributions are displayed. In **c**, the Maxwell–Boltzmann fit is depicted, and nonlinear fits at the exponential tails are also drawn (*c*: the normalized velocity, *α, β*: the fitting parameters)

The particle dynamics during applying the thermostat is explained in detail by showing particle-velocity distributions. Figure 7a, b shows the velocity distributions in each *x*-, *y*-, and *z* direction at the end of the excitation phase (20 ms) and at the end of the non-excited phase between excitations (80 ms) with Gaussian fits, respectively, and Fig. 7c shows the velocity-magnitude distribution with a Maxwell–Boltzmann fit. There is no remarkable difference between the distributions in different directions (Fig. 7a, b). Thus, the particles can be randomly excited in 3D by the magnetic thermostat. In addition, the velocity distribution at the end of the excitation phase has a higher population at larger velocity (Fig. 7a), due to the effect of 20 ms of a continuous external magnetic field. After the non-excited phase for 80 ms (Fig. 7b), the velocity distribution gets close to the Gaussian fit as the kurtosis *k* and the skewness *s* of the distribution near 0. It can also be seen in Fig. 7c that the high-energy tail decreases at the end of the non-excited phase. That is because fast particles collide with others randomly, and their kinetic energies are absorbed in the bulk of the particles. Therefore, three factors contribute to the realization of homogeneously and randomly excited particles in this system: the first factor is the magnetic interaction between particles, which attunes magnetic energies in particles and alleviates the preference of the external magnetic field–direction; the second factor is the switching from pair to pair of electromagnets to excite the particles in the long term isotropically; third is random collisions between particles, which absorb kinetic energies of excessively excited particles.

In conclusion, a numerical calculation method based on the DEM was developed to simulate a granular gas excited in microgravity by a thermostat utilizing a varying magnetic field. The presented calculation method considers magnetic interactions between the constituent particles. By analyzing the simulation results together with the experiment, it was demonstrated that three factors contribute to realizing homogeneously and randomly distributed particles in this thermostat: the first factor is the magnetic interaction between particles, second is switching from pair to pair between the electromagnets, and third is random collisions between particles in the short time window between each excitation. The simulations can reproduce the experimental results, and the calculation method allows optimizing the experimental setup and the condition before initiating the next experiment. The numerical and experimental results indicate that the thermostat is able to control the homogeneity and the excitation level of the granular gas without using any mechanical systems, just by changing the magnetic-excitation parameters. It is expected that this system can be useful for various future granular gas experiments performed in microgravity.

## Methods

A 3D DEM, based on the modified hard-sphere contact model,^32,33^ is performed to simulate the magnetically excited particles. The equation of motion for the *i*th particle can be represented as Eq. (1)

1 $$m_i{\ddot{\mathbf x}}_{\mathbf{i}} = \mathop {\sum}\limits_j {{\mathbf{F}}_{ij}}{,}$$

where *m*, **x**, and **F** are the particle mass (1.86 × 10^−5^ kg), the positional coordinate (*x, y, z*), and the external force, respectively. The subscript *j* represents a type of force applied to the *i*th particle. The velocity and position of the *i*th particle at each time can be calculated by solving Eq. (1) repeatedly with the short time step (1.0 × 10^−5^ s) using the Runge–Kutta method. When the particles collide with each other, the effect is calculated using the modified hard-sphere model.^32^ During the collision calculation, the coefficient of restitution *ε* is set to 0.55 for particle–particle collision, and 0.9 for particle–cell collision, respectively. These parameters were directly measured from the experiment.^2^ In this calculation, the air drag **F**_*a*_ is neglected due to the low pressure,^2^ and the magnetic force **F**_*m*_ is considered as external force by inserting Eq. (2) to the right-hand side of Eq. (1)^34^

2 $${\mathbf{F}}_{\mathbf{m}} = \left( {{\mathbf{p}}_i \cdot \nabla } \right){\mathbf{B}}_i{.}$$

Here, ***p*** and ***B*** are the magnetic dipole moment in the *i*th particle, and the magnetic flux density at the *i*th particle position, respectively. We assume that one magnetic dipole moment ***p*** is induced in the particle when that is subject to the magnetic field, and the magnetic dipole moment ***p*** can be represented by Eq. (3)^34^

3 $${\mathbf{p}}_i = \frac{{4\pi }}{{\mu _0}}\frac{{\mu _i - 1}}{{\mu _i + 2}}R_i^3{\mathbf{B}}_i$$

where *μ*_0_, *μ*_*i*_, and *R*_*i*_, are the magnetic permeability of free space (1.25 × 10^−6^ H/m), the relative magnetic permeability of the *i*th particle (10,000), and the *i*th particle radius (0.8 mm), respectively. The direction of the induced magnetic dipole moment ***p*** is the same as that of the magnetic field. Therefore, the magnetic force causes mostly translational motions of particles in this case. The external magnetic flux density field ***B***_external_ created by the electromagnet is calculated using the finite difference method to reproduce the experimental magnetic field. Two electromagnets aligned diagonally are turned on and the others are turned off at one point in time, and four kinds of the magnetic fields ***B***_external_ are required respectively. In DEM calculation, the calculated fields ***B***_external_ are turned on and off in the same order of the experimental procedure, and the magnetic flux density at the particle position is used to calculate Eqs. (2) and (3). As mentioned in the section “Introduction”, such high permeability gives the particles very low remnant magnetization as soon as the magnetic field is turned off. The simulation, therefore, ignores such long-range interactions when the magnetic field is turned off.

The magnetic particles are affected by the external magnetic field ***B***_external_ from the electromagnets and by the magnetically polarized particles in the field. Therefore, the magnetic flux density at the *i*th particle position is modified as Eq. (4)

4 $${\mathbf{B}}_i = {\mathbf{B}}_{\mathrm{external}} + \mathop {\sum}\limits_{k = 1,i \ne k}^N {{\mathbf{B}}_{ki}}{.}$$

Here, *N* is the particle number (3000). The first term and the second term on the right-hand side of Eq. (4) are the magnetic field generated by the electromagnets and that from the magnetic dipole moment generated in the *k*th particle. The magnetic flux density ***B***_*ki*_ from the *k*th particle is given by Eq. (5)^25^

5 $${\mathbf{B}}_{ki} = \frac{{\mu _0}}{{4\pi }}\left( {\frac{{3{\mathbf{p}}_k \cdot {\mathbf{r}}_{ki}}}{{\left| {{\mathbf{r}}_{ki}} \right|^5}}{\mathbf{r}}_{ki} - \frac{{{\mathbf{p}}_k}}{{\left| {{\mathbf{r}}_{ki}} \right|^3}}} \right){,}$$

where ***r***_*ki*_ is the positional vector from the *k*th to the *i*th particle. The modified magnetic flux density ***B*** is determined by solving Eqs. (3–5) simultaneously at each time step, and the magnetic force affected by the external magnetic field from the electromagnets and the magnetically polarized particles can be calculated by substituting the calculated ***p*** and ***B*** into Eq. (2).

**Fig. 8:**
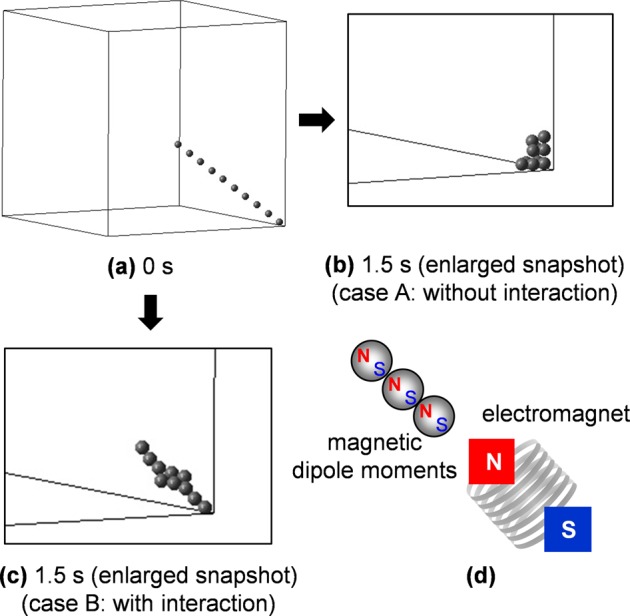
Calculated motions of ten particles in cases A (without magnetic interactions) and B (with magnetic interactions). **a** The particles are placed initially as aligned in a straight line at the sample cell corner and are attracted toward the corner by the external magnetic field in **b** case A and **c** case B. The particle chain is formed in case B due to magnetic dipole moment interactions, as shown in **d** the schematic

In this paper, two cases of the magnetic force are considered: (case A) neglecting magnetic particle–particle interactions, and (case B) including the interactions. Figure 8 shows the calculation results of a simple model in cases A and B to show the effect of considering the magnetic interactions between particles. Ten particles are initially placed in the sample cell (Fig. 8a). When only one pair of electromagnets is turned on during the whole calculation, the particles are attracted toward the corner in both cases A (Fig. 8b) and B (Fig. 8c). After 1.5 s of applying the magnetic field, the magnetic dipole moments created in particles interact with each other (Fig. 8d) and their chain is formed in case B (Fig. 8c), which is the typical behavior of magnetized particles,^25^ while particles are individually attracted and are stuck at the corner in case A (Fig. 8b).

In an actual calculation model to reproduce the experiment, all particles are randomly placed in the sample cell (5 × 5 × 5 cm^3^), and those are agitated by rounds of the magnetic excitations for 10 s. To reduce the calculation load, the whole calculation domain is divided in 3D into small cubic domains, with each side length five times larger than the particle diameter, and the magnetic interactions of particles within the same domain or neighbor domains are considered.

### Reporting summary

Further information on research design is available in the Nature Research Reporting Summary linked to this article.

## Supplementary information

Supplementary Movie 1.

Supplementary Movie 2.

Supplementary Movie 3.

Supplementary Information file.

Reporting Summary

## Data Availability

The data and the codes that support the findings of this study are available from the corresponding author upon request.

## Supplementary information

**Supplementary Information** accompanies the paper on the *npj Microgravity* website (10.1038/s41526-019-0079-y).
