# Supplementary material for: Magnetic excitation of a granular gas as a bulk thermostat
Source: NPJ Microgravity. 2019 Aug 13;5:19. doi: 10.1038/s41526-019-0079-y (PMC6692374; doi:10.1038/s41526-019-0079-y)
Supplement: Supplementary file 4 — Supplementary Information file. [file 41526_2019_79_MOESM4_ESM.pdf]

Supplementary Movie 1

Calculated motion of agitated particles in microgravity in case of neglecting magnetic interactions (excitation phase: 20 ms-on and 80 ms-off for 10 s, cooling phase: 5 s, playback speed: 0.4 times)

Supplementary Movie 2

Calculated motion of agitated particles in microgravity in case of considering magnetic interactions (excitation phase: 20 ms-on and 80 ms-off for 10 s, cooling phase: 5 s, playback speed: 0.4 times)

Supplementary Movie 3

Observed motion of agitated particles in the experiment in microgravity (excitation phase: 20 ms-on and 80 ms-off for 10 s, cooling phase: 5 s, playback speed: 0.4 times)
